# Deep learning-based phenotyping for genome wide association studies of sudden death syndrome in soybean

**DOI:** 10.3389/fpls.2022.966244

**Published:** 2022-10-21

**Authors:** Ashlyn Rairdin, Fateme Fotouhi, Jiaoping Zhang, Daren S. Mueller, Baskar Ganapathysubramanian, Asheesh K. Singh, Somak Dutta, Soumik Sarkar, Arti Singh

**Affiliations:** ^1^ Department of Agronomy, Iowa State University, Ames, IA, United States; ^2^ Department of Mechanical Engineering, Iowa State University, Ames, IA, United States; ^3^ Department of Computer Science, Iowa State University, Ames, IA, United States; ^4^ Department of Plant Pathology and Microbiology, Iowa State University, Ames, IA, United States; ^5^ Department of Statistics, Iowa State University, Ames, IA, United States

**Keywords:** stress phenotyping, disease quantification, object detection, foreground detection, ROC analysis, image-based phenotyping, deep learning

## Abstract

Using a reliable and accurate method to phenotype disease incidence and severity is essential to unravel the complex genetic architecture of disease resistance in plants, and to develop disease resistant cultivars. Genome-wide association studies (GWAS) involve phenotyping large numbers of accessions, and have been used for a myriad of traits. In field studies, genetic accessions are phenotyped across multiple environments and replications, which takes a significant amount of labor and resources. Deep Learning (DL) techniques can be effective for analyzing image-based tasks; thus DL methods are becoming more routine for phenotyping traits to save time and effort. This research aims to conduct GWAS on sudden death syndrome (SDS) of soybean [*Glycine max* L. (Merr.)] using disease severity from both visual field ratings and DL-based (using images) severity ratings collected from 473 accessions. Images were processed through a DL framework that identified soybean leaflets with SDS symptoms, and then quantified the disease severity on those leaflets into a few classes with mean Average Precision of 0.34 on unseen test data. Both visual field ratings and image-based ratings identified significant single nucleotide polymorphism (SNP) markers associated with disease resistance. These significant SNP markers are either in the proximity of previously reported candidate genes for SDS or near potentially novel candidate genes. Four previously reported SDS QTL were identified that contained a significant SNPs, from this study, from both a visual field rating and an image-based rating. The results of this study provide an exciting avenue of using DL to capture complex phenotypic traits from images to get comparable or more insightful results compared to subjective visual field phenotyping of traits for disease symptoms.

## 1 Introduction

Sudden death syndrome (SDS) is a fungal disease in soybean [*Glycine max* L. (Merr.)] caused by *Fusarium virguliforme* in North America (Aoki et al., 2003). In recent years, another known pathogen causing SDS *Fusarium brasiliense* has also been identified within the U.S (Wang et al., 2019). The pathogens known to cause SDS have been identified in North America, South America, and Africa (Wang et al., 2019). SDS first develops in the roots and as the fungus infects the roots it releases a toxin within the plant (Hartman et al., 2015). During the reproductive stages, foliar symptoms can begin to develop in infected plants (Hartman et al., 2015). Leaves start showing chlorotic spots between the veins and the spots continue to expand and grow until the tissue dies (Hartman et al., 2015). In a meta-analysis of the relationship between yield and SDS infection, it was found that at the R6/R5 reproductive stage, for every unit of foliar index (0-100) increase the yield decreased by 0.5% (Kandel et al., 2020). At the highest level of disease severity this would be a 50% yield reduction (Kandel et al., 2020). Between 2015-2019 the estimated yield loss for 28 states in the U.S. and Canada due to SDS was 189 million bushels of soybean (Bradley et al., 2021), demonstrating the importance of this disease to producers and economy.

The prevalence and economic impact of SDS make it a key breeding target in cultivar development programs as the combination of in-season fungicide application with resistant cultivars provide better management of SDS than in-season fungicide application alone (Kandel et al., 2016). Studies have shown that there are few options for managing SDS with agricultural practices (Xing and Westphal, 2009; Hartman et al., 2015). Weather conditions, such as rainfall, can impact the prevalence of SDS (Leandro et al., 2013). Due to limited solutions to prevent SDS through agricultural practices and unpredictable yearly variable weather conditions, the most promising solution for preventing SDS infection is the development of resistant cultivars (Singh et al., 2021). However, only a few lines have been developed that are moderately resistant to SDS (Rodriguez et al., 2021). In order to develop SDS resistant soybean cultivars, higher throughput and more precise phenotyping is necessary to identify resistant accessions in breeding programs. Additionally, more information needs to be available to breeders regarding molecular markers linked to genetic loci or quantitative trait loci (QTL) controlling SDS.

There are around 104 SDS QTL, identified in bi-parental RIL populations, reported on Soybase ([Dataset] et al., 2010), along with an additional 84 SDS QTL identified using genome-wide association studies (GWAS) (Wen et al., 2014; Chang et al., 2018). GWAS are extremely useful to investigate the genetic background of more complex traits (Zhu et al., 2008; Tibbs Cortes et al., 2021). Useful insights have been generated through GWAS and genome-wide epistatic studies (GWES) for multiple diseases and stress traits in soybean such as *Sclerotinia* stem rot (Moellers et al., 2017), Charcoal rot (Coser et al., 2017) and iron deficiency chlorosis (Assefa et al., 2020). Significant single nucleotide polymorphism (SNP), SNP-SNP interactions, and QTL associated with SDS resistance have been reported in soybean using GWAS (Wen et al., 2014; Zhang et al., 2015; Chang et al., 2016; Swaminathan et al., 2019). Most of these studies utilize visual rating scales, such as disease severity, as described by Zhang et al. (2015). Visual ratings are time-consuming and can be unreliable due to inter-rater and intra-rater variability (Akintayo et al., 2018; Singh et al., 2021b). Several of these challenges have been addressed with the use of Machine Learning (ML) methods, as they enable more reliable high-throughput phenotyping systems (Singh et al., 2016; Moellers et al., 2017; Coser et al., 2017; Singh et al., 2021). ML methods also allow researchers to use large datasets without increasing the time needed to phenotype crop traits compared to manual methods (Singh et al., 2016; Singh et al., 2018; Assefa et al., 2020). Different ML algorithms have been studied in analyzing soybean phenotypes, such as Support Vector Regression (SVR) (Yoosefzadeh Najafabadi et al., 2021), Random Forest (RF) (Yoosefzadeh-Najafabadi et al., 2022), and K-Nearest Neighbors (KNN) (Naik et al., 2017). These recent studies show ML methods are successful in analyzing the numerical data in GWAS studies compared to previous statistical methods. However, extracting phenotyping features directly from the digital data (images and videos) is more challenging and cannot properly happen through the classical ML methods.

Deep Learning (DL), as a subset of ML methods relying on the artificial neural networks recently could achieve promising results in extracting higher-level features from images (Singh et al., 2018; Falk et al., 2020; Singh et al., 2021b). Also, several studies in agriculture show that DL frameworks can successfully extract phenotypic information from images of leaves (Pires et al., 2016; Zhang et al., 2017), roots (Falk et al., 2020), stem (Nagasubramanian et al., 2019), pods (Riera et al., 2021), nodules (Jubery et al., 2021) and canopies (Parmley et al., 2019a; Parmley et al., 2019b; Tetila et al., 2020). Among DL-based computer vision tasks, object detection aims to detect and localize the instances in each image which, in practice, is utilized in agriculture applications such as crop monitoring, disease detection and pest detection (Zhao et al., 2019; Wani et al., 2021; Chen et al., 2021; Pratama et al., 2020) deep. Object detection methods can be categorized as one-stage or two-stage detectors. The region proposal stage in a two-stage object detector should be applied before training that typically makes these methods time-consuming (Girshick, 2015; Dai et al., 2016; He et al., 2017; Cai and Vasconcelos, 2018). Moreover, the efficiency of two-stage method in real-world scenarios is questionable, especially while using edge devices, e.g., cameras and smartphones.

Therefore, one-stage object detectors have been preferred in the cases with time constraints as they can yield desirable accuracy faster by removing the intermediate task of proposing regions. This becomes particularly more important where the eventual goal is on-spot disease monitoring for scouting or disease ratings. Among these methods, YOLO and SSD are quite popular one-stage object detectors especially considering the speed and accuracy trade-off (Liu et al., 2016; Redmon and Farhadi, 2017; Fu et al., 2017). One of the obstacles to achieve top accuracy in one-stage methods is their weakness in recognizing the class imbalance in some datasets (Oksuz et al., 2020). Such issues can be alleviated by using architectures such as the RetinaNet  (Lin et al., 2017a).

In the RetinaNet method, a loss function is defined (*focal loss*) to overcome the imbalanced class distribution problem (Lin et al., 2017a). If the confidence in a correct class increases, the scaling factor for this loss function is pushed to zero and vice versa. Consequently, the RetinaNet algorithm focuses on the more problematic examples by increasing their contribution to train the model rather than focusing on the easy examples. The feature pyramid network in the RetinaNet model merges semantically more vital features with features from previous layers (Lin et al., 2017b). Besides Nguyen et al. (2020), in their review paper shows that RetinaNet method demonstrates promising results for small object detection. In agriculture applications especially with imbalanced data sets, such as disease and insect classification, the RetinaNet object detector has been utilized to overcome this common challenge (Sales et al., 2021; Correa et al., 2021; Bao et al., 2022).

Success of ML-based plant stress phenotyping enabled the emergence of the paradigm of automated identification, classification, quantification, and prediction (ICQP) of plant stresses (Singh et al., 2016). For example, the study by Zhang et al. (2017) involved identification and classification of iron deficiency chlorosis (IDC) in canopy images of soybeans that were then used in a GWAS. However, in many data sets, only an individual plant organ (e.g., leaf or stem) is present in each image (Pires et al., 2016; Tetila et al., 2020). This makes the object detection part far less challenging than data sets containing (part of) the plant canopy captured directly from the field. Therefore, little research has been done leveraging object localization and classification for plant stress phenotyping. Tran (2019). detected diseased leaves in the soybean canopy. However, the disease severity for each leaf or leaflet was not quantified.

**Figure 1:**
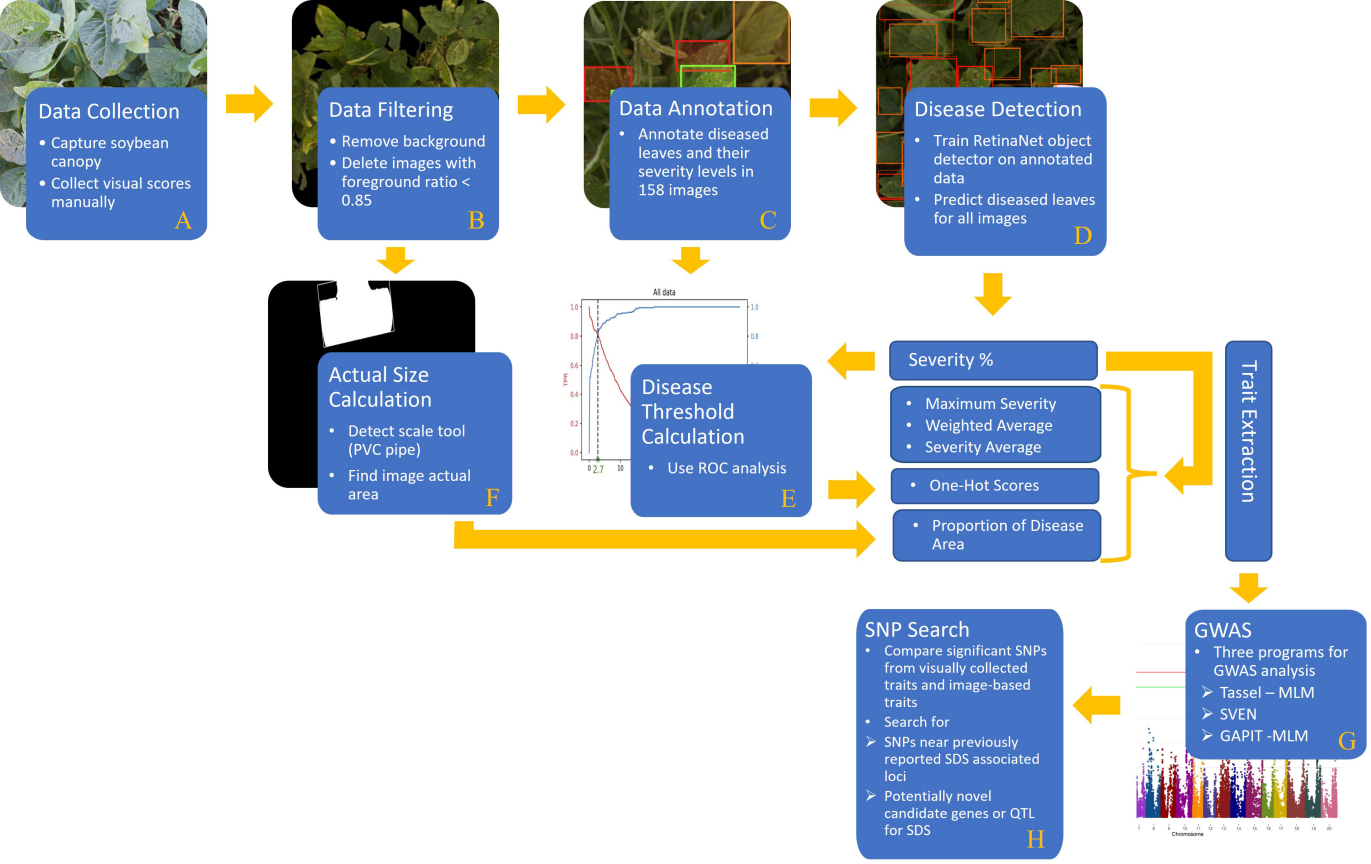
A comprehensive flowchart of the steps of our proposed method: **(A)** data collection, **(B)** data filtering, **(C)** data annotation, **(D)** disease detection, **(E)** disease threshold calculation, **(F)** actual size calculation, **(G)** GWAS, and **(H)** SNP search.

The objective of this study is to present the effectiveness and accuracy of a DL-based model in detecting and quantifying SDS disease severity. In this regard, GWAS analysis was applied on DL-based SDS disease ratings and was compared to that using visual/manual ratings done in the field. In summary, our proposed framework was built using the following steps (**Figure 1**):

(A) a comprehensive data set containing soybean canopy from two different locations was collected in two consecutive years. This dataset contains a diverse population of 479 soybean accessions. (B) Foreground detection computer vision technique was applied to remove the background of images as well as compute the foreground ratio in each image. (C) In order to train an object detector (RetinaNet), some images were labeled by an expert team in which each diseased leaflet was classified based on its disease severity level. (D) The RetinaNet architecture was selected due to the fast training and high-precision even using an unbalanced dataset. Once the model is trained, disease severity levels of soybean leaves (specifically, leaflets) can be localized and classified efficiently. (E) The output of the DL model goes through a few post-processing steps (i.e. ROC analysis) to generate the desired phenotypic traits. (F) Moreover, the actual size of the images was computed by detecting a size scale tool (PVC pipe) in the images. Then, the proportion of disease area in the images was calculated and considered as another phenotypic trait. (G) GWAS was performed with both the manually collected disease scores and the machine generated ones. All traits were evaluated with three different statistical softwares for GWAS analysis. (H) Significant single nucleotide polymorphisms (SNPs) are reported and compared to previously reported SDS QTL and significant SNP from association and linkage mapping studies. This validated the use of machine-based traits compared to the traditional manually collected traits. Then novel loci of SDS resistance were explored from the GWAS results. Results based off the DL-traits showed agreement with past studies as well as potential novel sources of resistance which in turn proves the practicality and reliability of the DL-based model for disease phenotyping coupled with genetic studies.

## 2 Material and methods

### 2.1 Plant material and field trials

A diverse population of 479 soybean accessions was studied in this research, and is referred to as GWAS panel. It included 473 plant introduction (PI) accession lines representing a mini-core collection of the United States Department of Agriculture (USDA) early maturity soybean germplasm collection with checks of various levels of resistance including 92Y60 (susceptible), 92Y83 (resistant). The GWAS panel was planted at Ames and Muscatine, IA, both in 2015 and 2016. Each plot consisted of two rows of 1.5 meters length with 76.2 cm between rows. All field trials were planted as a randomized complete block design with 2 replications. Before planting, soybean seeds are mixed with sorghum grain infested with *Fusarium virguliforme* for disease inoculation. Artificial irrigation was provided to help in disease development.

### 2.2 Genotyping

The genotypic data of the PI lines was previously prepared *via* the SoySNP50K BeadChip (Song et al., 2013; Song et al., 2015) and was retrieved from SoyBase (Grant et al., 2010). There are 42,195 SNPs within this panel. Using the TASSEL 5 filtering function, sites with a minor allele frequency (MAF) less than 5% and 1% are filtered out and minor SNP states were removed (Bradbury et al., 2007). Separate GWAS analysis were conducted for MAF of 5% and 1%. Numerical imputation was performed using k-nearest neighbors, with k equal to five and Euclidean distance, with the TASSEL 5 Numerical Impute function (Bradbury et al., 2007). Numerical genotypic files were exported from TASSEL 5 for use across all three programs (Bradbury et al., 2007).

### 2.3 Image acquisition and filtering

To develop a model for identifying SDS severity, a total of 3161 images were collected immediately after visual ratings at the R6 stage in three of the environments: Ames 2015, Muscatine 2015, and Muscatine 2016 (Fehr et al., 1971). Ames 2016 was dropped from further analysis since only visual scores were collected in that location and imaging was not done. Images were taken by following the protocol as described previously (Zhang et al., 2017). In addition to the previously described protocol, a PVC tee pipe with a width of 6.35 cm was held near the canopy while imaging to be used for scale. In our dataset, we only imaged the area of the canopy showing the most severe symptoms; and the edge of the plots were avoided for imaging. After collection of the image dataset, images were reviewed for picture quality. Image-based traits assume most of the image foreground is plant canopy. Therefore, images were evaluated based on their foreground ratio or the ratio of pixels that are canopy to the number of pixels in the whole photo. A total of 2772 images remained for analysis after filtering. Please see **Figure S1** in the Supplementary.

#### 2.3.1 Actual image area calculation

**Figure 2:**
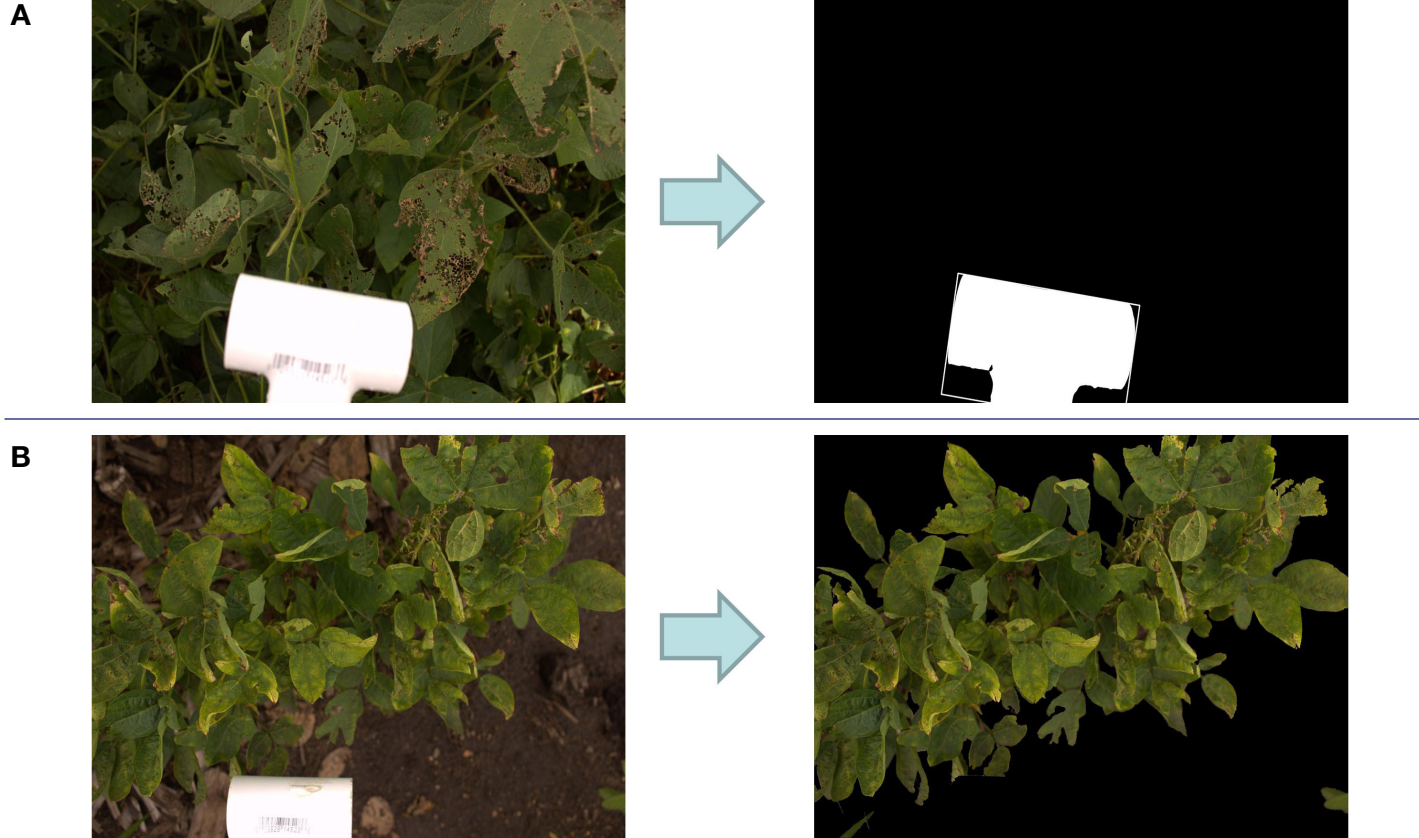
**(A)** Detection of the size scale tool (PVC fitting pipe) in a sample image which results in finding the actual image size. **(B)** Removing background of a sample image for foreground ratio calculation.

As mentioned above, in all images, a 6.35 cm length PVC fitting was used as a reference object to compute the images’ actual sizes. Image processing steps were implemented to detect this white object in the image. First, the image was converted to a black and white image with one channel or gray scale image. Then, a threshold gray color (pixel value = 230) was considered to binary classify the image pixels to black (pixel value = 0) and white pixels (pixel value = 255). For each pixel in the gray scale image, if the pixel value is more than the threshed it was converted to white pixel and otherwise black pixel. Afterward, the biggest contour of white pixels was selected from the detected white pixels in the image. Finally, a rectangle was fitted to this contour. **Figure 2A** shows an example of the result of this image processing algorithm. The length of this rectangle is considered as the length of the PVC fitting. Therefore, if this length in the image consists of *P* pixels, the image size per pixel would be 6.35/*P*.

For some images (7% of total images), the detected white contour was not accurate likely because of two reasons: (a) either the brightness of some images was high resulting in a lot of scattered white pixels in the images or (b) the orientation, and position of the PVC fitting are different from other images. Therefore, for computing the actual area of these images, ImageJ software was used. In this software, the number of pixels in the length of the PVC could be calculated manually by selecting the start and end pixels of the PVC length in the image.

#### 2.3.2 Computing foreground ratio

For computing the foreground ratio, images were converted to HSV (Hue, Saturation, Value) color space and pixels colors in the range of HSV values from (20, 32, 20) to (100, 255, 255) were kept while the remaining pixels were dropped. The contours that contain all of the pixels in the defined green range were considered as image foreground as shown in **Figure 2B**. The foreground ratio was computed by dividing the foreground portion of image on the whole image. Images with a foreground ratio of less than 85% were selected to be manually evaluated for removal from the dataset. **Supplementary Figure S1** shows the distribution of foreground ratio in the dataset. A total of 398 images had below the 85% foreground ratio and were manually evaluated. Manual filtering left a total of 2772 images for further evaluation (**Supplementary Table S1**). Removed images include those that were mostly soil (**Supplementary Figure S2A**), those that were of Color Checker Charts (**Supplementary Figure S2B**), and images that signified the end of a row (**Supplementary Figure S2C**).

### 2.4 Deep learning approach

#### 2.4.1 Dataset labeling and preprocessing

Five different classes were used to define a single leaflet’s disease state in the image. The severity classes are Healthy, Severity 1, Severity 2, Severity 3, and Severity 4. The class of Healthy is defined as a leaflet without any SDS disease symptoms, and this class is dropped to focus on susceptibility to SDS and the variability in severity of SDS.

A total of 158 images were selected as a subset from the whole data set for manual annotation and model development. The selected images were chosen accurately to represent the variety of disease severities - see section on disease phenotyping. In total, 2603 bounding boxes manually annotated by an expert team through LabelBox software (**Table 1**).

**Table 1:** Breakdown of number of bounding boxes for each of the four severity classes.

Class Name	Severity 1	Severity 2	Severity 3	Severity 4	Total
**Number of BB**	1208	665	294	436	2603

**Figure 3:**
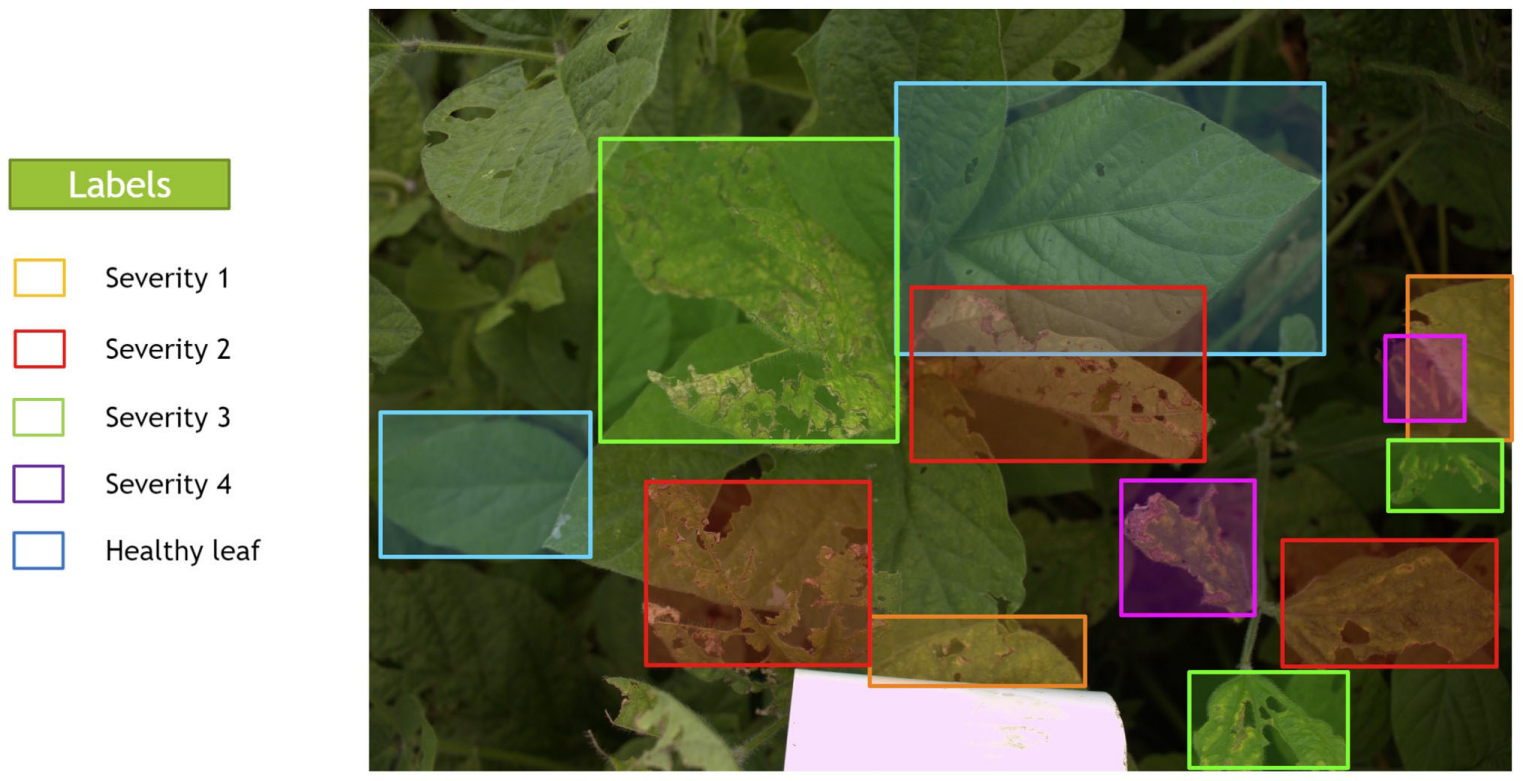
A sample image that was manually annotated by an expert with bounding boxes thorough LabelBox. Each severity level class was assigned a different color of box.

In LabelBox, different colors were assigned to each of the classes’ bounding boxes in the images, which were blue, orange, red, green, and purple for Healthy, Severity 1, Severity 2, Severity 3, and Severity 4, respectively, as shown in **Figure 3**. Further, the dataset was randomly divided into training, validation, and testing sets with the ratio 80%, 10%, and 10%, respectively. The model will be evaluated on the validation set after each epoch during training, similar to other DL methods. The unseen test set (holdout dataset) is kept unseen for reporting the final results.

#### 2.4.2 Deep learning model

RetinaNet is a one-stage object detector using a special loss (*focal loss*) which is meant to address the foreground-background class imbalance problem as it happens in our labeled dataset (Lin et al., 2017a) (**Table 1**). Generally, the RetinaNet model is composed of a backbone network and two task-specific subnetworks. The backbone network is devised of any convolutional backbone networks like Residual Networks (ResNet), Visual Geometry Group (VGG), and Densely connected convolutional Network (DenseNet) to extract the convolutional feature map of all the entire input images. Afterward, the Feature Pyramid Network (FPN) (Lin et al., 2017b) completes the backbone and extracts the different scales of the same dimension features. The first subnetwork classifies the objects by applying a convolutional object classification, which performs on the backbone’s output. Second subnetwork using to locate the objects by executing convolutional bounding box regression. Further details regarding the RetinaNet model can be found in **Supplementary Material section 1**.

In this work, in order to increase the number of training samples, the training data was augmented by rotating (90 degrees), flipping and changing illumination. Moreover, we added two regularization methods to the RetinaNet model to prevent the overfitting problem, which were early stopping and dropout. By applying the early stopping method, the model is trained until the validation loss does not decrease for three consequence epochs (which is the point that the training loss continues to drop and the model starts overfitting). Also, using dropout, the model could simulate having a different number of network architectures for training procedures by randomly dropping out some nodes from dense neural network layers.

Two well-known metrics were used, Intersection over Union (IOU) and Mean Average Precision (mAP) to evaluate our results. IOU can be determined by equation 1 by considering the ground truth and model predicted bounding boxes. This metric is reported True Positive (*TP*), False Positive (*FP*), False Negative (*FN*) bounding boxes by considering a special threshold (in this work 0.5). If the predicted and ground truth bounding boxes have IoU more than 0.5, the predicted bounding box is denoted as *TP*. On the other hand, if IoU of predicted and ground truth bounding boxes is less than the defined threshold, the predicted bounding box is indicated as *FP* and the ground truth bounding box is denoted as *FN*.


 (1) $$IoU=\frac{Area\;of\;Overlap\;of\;Predicted\;and\;Labeled\;Bounding\;Boxes}{Area\;of\;Union\;of\;Predicted\;and\;Labeled\;Bounding\;Boxes}$$

Before discussing the DL model evaluation metrics, a review of the definitions of recall and precision is also required. The *Precision* and *Recall* in DL methods are defined in equation 2 using the extracted *TP*, *FP*, and *FN*.


(2) $$Precision=\frac{TP}{TP+FP},\;Recall=\frac{TP}{TP+FN}$$

Taking into account the definitions of Precision and Recall, equation 3 defines Average Precision (*AP*) which is the area under the precision-recall plot for each class.


(3) $$AP=\int_{0}^{1}p\left(r\right)dr$$

Then, the mean average precision (equation 4) would be the mean of *AP*s over a set of queries (*M* is the total number of queries).


(4) $$mAP=\frac{1}{M}\sum_{m=1}^{M}AP\left(q\right)$$

Another evaluation metric in order to report the object detector performance is *F*_1_ score. This metric also represents the harmonic mean of precision and recall values as follows:


(5) $$F_{1}=2\frac{Precision.Recall}{Precision+Recall}$$

Accuracy is also a well-known metric in classification, which is computed as follows:


(6) $$Accuracy=\frac{TP+TN}{TP+FP+TN+FN}$$

Besides accuracy, mAP, and *F*_1_ score metrics, we report our results based on the Matthews Correlation Coefficient (MCC) metric, which is not affected by the unbalanced datasets issue. MCC is a method of computing the Pearson product-moment correlation coefficient (Powers, 2020) between actual and predicted values, which is defined as follows:


(7) $$MCC=\frac{TP.TN-FP.FN}{\sqrt{\left(TP+FP\right).\left(TP+FN\right).\left(TN+FP\right).\left(TN+FN\right)}}$$

### 2.5 Phenotyping

#### 2.5.1 Manual

There were two traits manually collected in the field. Disease severity (DS) and disease incidence (DI) were taken through visual rating at the R6 stage (i.e., full seed) for all three retained environments. The disease severity was ranked by using a 0-9 scale: 0 indicates fully resistant, and 9 indicates most susceptible (Wen et al., 2014). Disease incidence is the percentage of plants in the plot showing leaf symptoms where 0% means no symptoms and 100% means all plants have leaves that show symptoms of SDS (Wen et al., 2014). The disease index (DX) is a metric calculated from the collected scores of disease severity and disease incidence. Equation 8 shows the calculation of disease index (Njiti et al., 2001; Wen et al., 2014; Kandel et al., 2020).


(8) $$Disease\;Index=DI*\frac{DS}{9}$$

#### 2.5.2 Image-based

Images processed through the RetinaNet network generated an output of bounding boxes with classifications for one of the four severity levels. This output was used to generate a severity percentage, which was then used to characterize several other traits. Severity Percentage is the number of pixels, of Severity X (where X is 1, 2, 3, or 4), in the images over the total number of pixels in the image (equation 9).


(9) $$Severity_{X}\%=\frac{Total\;Area\;of\;Bounding\;Boxes\;of\;Severity\;X}{Total\;Area\;of\;the\;Image}*100$$

For each image, the highest severity level that has value was considered the Maximum Severity. Proportion of Disease Area was calculated by taking the summation of the area of all bounding boxes and dividing it by the total area of the image as it is shown in equation 10. This represents the area of the image that is showing disease symptoms.


(10) $$Proportion\;of\;Disease\;Area=\frac{Total\;Area\;of\;all\;Bounding\;Boxes}{Total\;Area\;of\;the\;Image}$$

Two of the phenotypic traits extracted from the images consider the weighted value of each class. Each class is assigned the weight to its class number (i.e., Severity 1 was assigned a weight of 1, and Severity 2 was assigned a weight of 2, and so on). Weighted Average compares the weighted Severity Percentages to the summation of the overall scale (equation 11).


(11) $$Weighted\;Average=\frac{\left(Severity_{1}\%\times 1\right)+\left(Severity_{2}\%\times 2\right)+\left(Severity_{3}\%\times 3\right)+\left(Severity_{4}\%\times 4\right)}{1+2+3+4}$$

Severity Average considers the weighted score of each severity class divided by the total severity classification within the image. Severity Average is calculated in equation 12 as follows:


(12) $$Severity\;Average=\frac{\left(Severity_{1}\%\times 1\right)+\left(Severity_{2}\%\times 2\right)+\left(Severity_{3}\%\times 3\right)+\left(Severity_{4}\%\times 4\right)}{Severity_{1}\%+Severity_{2}\%+Severity_{3}\%+Severity_{4}\%}$$

Due to the distribution of Severity Average, a log-transformation was used. Exploration of the data revealed that some plots only contained healthy leaflets which leads to a Severity Average score of 0. These plots were dropped before log transformation. All results regarding Severity Average are reported using the log-transformed trait data. The correlation between manually collected traits and DL traits extracted from images was calculated using Pearson’s correlation.

#### 2.5.3 One-hot encoding

Another trait extracted from the images was a One-Hot severity score. Four binary digits *b*_1_ *b*_2_ *b*_3_ *b*_4_; which *b*_1_, *b*_2_, *b*_3_, and *b*_4_ binary values correspond to the occurrence of Severity 1, Severity 2, Severity 3, and Severity 4 in that image, respectively, is denoted as “One-Hot Encoding” in **Table 2**. For example, 1111 indicates the image has all of the severity levels. Therefore, finding an appropriate threshold (*T_sp_*) for Severity Percentage values can help us decide whether a specific severity actually exists in the image directly from the DL model outputs. If the Severity Percentage for the class *x* is more than this threshold (*Severity_x_*% ≥ *T_sp_*), the binary value will be 1; otherwise (*Severity_x_*%< *T_sp_*), it will be 0. Comparing this binary values with the threshold for all of the severity classes and concatenating them can give us the One-Hot encoded value.

**Table 2:** Conversions of concatenated One-Hot encoded values to One-Hot Score representing disease rating and severity classification to be used in GWAS analysis.

One-Hot Encoded	Disease Severity Classification	One-Hot Score (Disease Score)
1000	Low	1
1100	Low-Int	2
0100	Int	3
1110	Int-High	4
0110	Int-High	4
1010	Int-High	4
0010	High	5
1111	High-V. High	6
1101	High-V. High	6
1011	High-V. High	6
0111	High-V. High	6
0011	High-V. High	6
0101	High-V. High	6
1001	High-V. High	6
0001	V. High	7
0000	NIL	NIL

**Figure 4:**
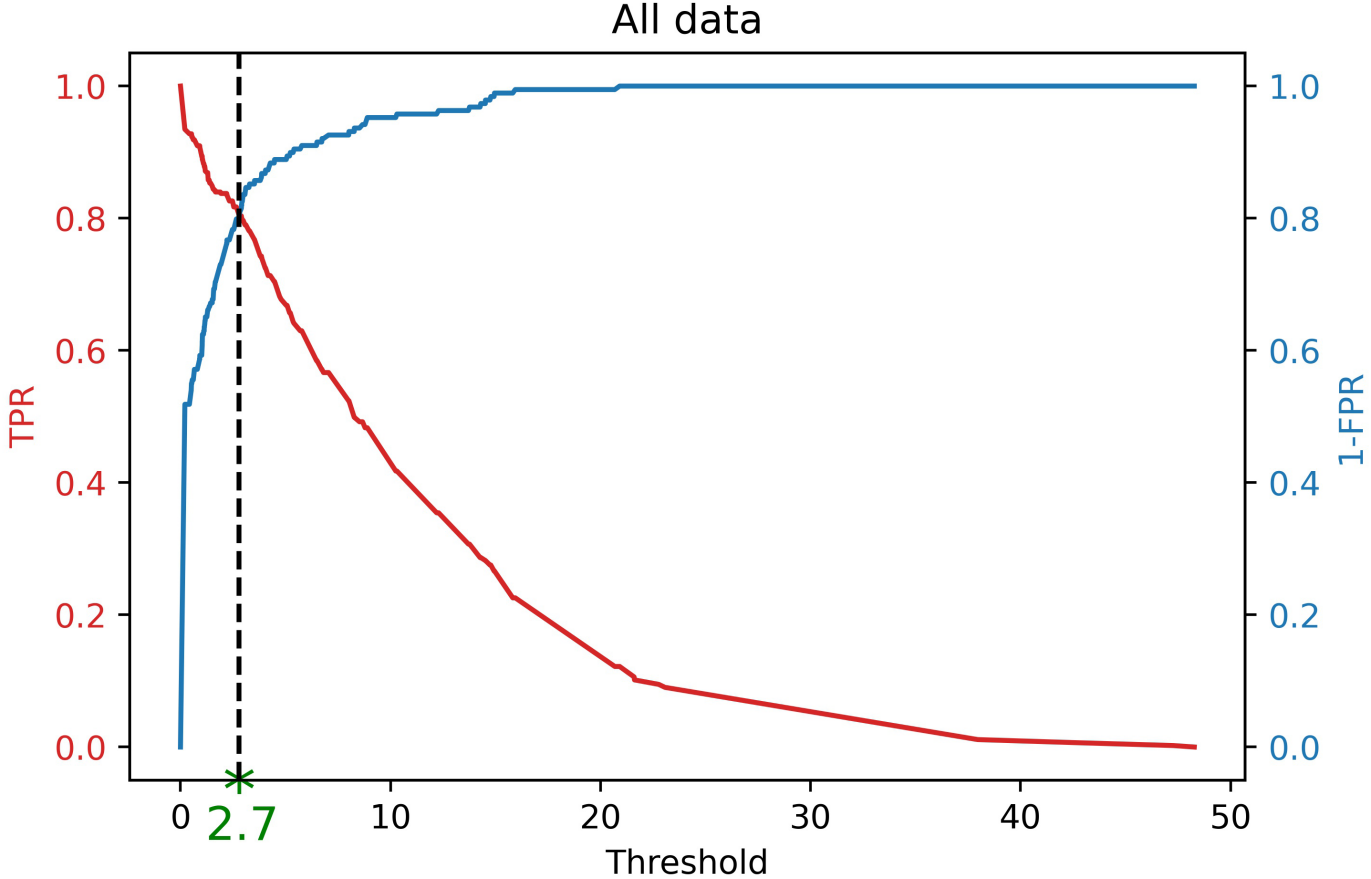
Finding optimal threshold (*T_sp_* = 2.7) of DL results for finding One-Hot encoding using ROC analysis. If the severity percentage value for a specific severity level is more than this threshold, it is encoded as 1, otherwise,0. Therefore, this encoded value shows the occurrence of that severity level in an image based on the DL results.

Note that the labeled bounding boxes are a proper resource to guarantee that a severity level occurs on a canopy in an image. Therefore, considering the labeled data, the ground truth One-Hot encoded values can also be extracted for each image. Taking into account these One-Hot encoded values as well as the Severity Percentages of the model output of the labeled data, the optimal threshold was computed by using Receiver Operating Characteristic (ROC) analysis (Fawcett, 2006). Based on ROC analysis, the optimal threshold is a cut-off point where the True Positive Rate ( $TPR=\frac{TP}{TP+FN}$ or *Sensitivity*) is high and False Negative Rate ( $FNR=\frac{FN}{FN+TP}$ or 1− *Specificity*) is low (Zhu et al., 2010). **Figure 4** shows the best threshold (*T_sp_ =* 2.7) that the object detector classifier offers on our dataset which is a point where *Sensitivity* and *Specificity* curves intersect. Theoretically, this threshold can be computed as follows (equation 13).


(13) $$T_{sp}=\frac{\sqrt{TPR\times FPR}-FPR}{TPR-FPR}$$

Considering this threshold, the One-Hot encoded binary values for other images can be extracted. If the Severity Percentage was greater than the threshold then that severity class was assigned a value of 1 for that image, if the Severity Percentage was less than the threshold it was assigned a value of 0. Then, the four numbers generated by this evaluation, from all the classes were concatenated into a One-Hot encoded value.

**Figure 5:**
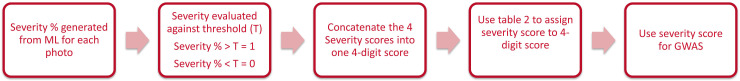
Workflow of generation of One-Hot Score and conversion to disease score used in GWAS analysis.

The One-Hot encoded value was then compared to the chart in **Table 2** to determine a disease rating (called “One-Hot Score”) for that image. The process of how One-Hot Scores are determined is displayed in the flow chart in **Figure 5**.

### 2.6 Genome-wide association analysis

#### 2.6.1 Statistical analysis

Lines were evaluated based on their best linear unbiased predictor (BLUPs) which is calculated for each trait using the statgenSTA package with the “lme4” engine. For each trait, BLUPs were generated within Ames 2015, Muscatine 2015, and Muscatine 2016. Calculation of BLUPs uses equation 14:


(14) $$Y_{ij}=\mu +G_{i}+R_{j}+\epsilon_{\left(ij\right)}$$

Where *Y_ij_* is the phenotypic value of the ith genotype in the jth replication, *μ* is the population mean, *G_i_* is the random genotypic contribution for the ith genotype, *R_j_* is the random block effect of the jth replication, and *ϵ_ij_* is the residual. Broad sense heritability (*H^2^*) was determined by equation 15:


(15) $$H^{2}=V_{G}/V_{P}$$

Where *V_G_* is genotypic variance and *V_P_* is phenotypic variance.

#### 2.6.2 Genome-wide association method

The GWAS analysis was performed with three different programs: Tassel 5 (Bradbury et al., 2007), GAPIT version 3 (Wang and Zhang, 2021), and SVEN (Li et al., 2022). Genotypic data was loaded into Tassel 5 and prepared as described in section 2.2. The genotypic data was used to calculate a kinship matrix with Centered IBS in Tassel 5, which was then exported for use in GAPIT. Principle component analysis (PCA) was then preformed with three components in Tassel 5. Model selection within GAPIT version 3 indicated the optimal number of components for PCA was no components, but still three were used to account for any familial or population structure. Within Tassel 5 the MLM model was used to find associations between SNPs and all 8 phenotypes (Bradbury et al., 2007; Zhang et al., 2010). The same data was supplied to GAPIT version 3 for analysis with the MLM model as well (Yu et al., 2006; Wang and Zhang, 2021). For both GAPIT and Tassel the MLM model was used which is described as:


(16) y=Xa+Zb+e

where *y* is a vector of phenotypic observations, *α* is a vector of fixed effects that includes the population structure, *b* is a vector that includes genetic effects defined by a kinship matrix, *X* and *Z* are design matrices, and *e* is the vector of residual effects. For both Tassel 5 and GAPIT version 3, a False Discovery Rate (FDR) correction was used. A threshold of p = 0.05 was used and the function qvalue from the package qvalue was used to calculate the FDR with this threshold (Storey et al., 2020). The third model we used was Selection of Variables with Embedded Screening (SVEN) (Li et al., 2022).

SVEN is a Bayesian method based on a hierarchical multi-locus model that controls for false discovery through prior regularization on the number of important makers. In order to find the important markers, SVEN starts from an empty set of markers, and repeatedly randomizes among the following moves: (a) add a potentially important marker, (b) remove a previously added marker whose importance may have been reduced with the discovery of better markers, and (c) swap a previously added marker with another potentially important marker. The randomization is done based on posterior importance probability. Employing these stochastic moves, SVEN rapidly identifies groups of markers with high posterior probabilities. Using the posterior probabilities of these groups, the marginal inclusion probability (MIP) of each marker was computed after accounting for the rest of the markers. The markers with MIP bigger than 0.5 are reported (Barbieri and Berger, 2004). We use the R-package bravo (Li et al., 2021) that has SVEN implemented. Because SDS in soybean is a complex trait (Hnetkovsky et al., 1996; Roy et al., 1997; Hartman et al., 2015), we set a relatively high prior shrinkage lambda = 20, and prior inclusion probability w ranging from 0.00051 to 0.00059 depending on the number of accessions tested in the environments following the suggestions of Li et al. (2022). Candidate gene search was done using the Genome Browser and the Genetic Map of SDS associated QTL on Soybase (Grant et al., 2010). Further details on methods used to search for candidate genes and SDS associated QTL for significant SNPs are available in the article by Brown et al. (2021).

## 3 Results

### 3.1 Phenotyping

Several traits were extracted from the image data that was processed through the RetinaNet network. Distribution values such as mean, standard deviation, and range can be seen in **Table 3** for each of the traits along with their broad sense heritability within each environment. Traits collected manually in the field had a heritability over 0.40 in all environments. DL traits extracted from images had lower heritabilities in Muscatine 2015 compared to the other two environments. Within Ames 2015 and Muscatine 2016 the heritabilities for DL associated traits were over 0.40 as well, except for Maximum Severity in Muscatine 2016 as it is shown in **Table 3**. There is a positive correlation within the manually collected traits and within the DL associated traits. Between the manually collected traits and DL associated traits there is a low correlation (**Figure S3**).

**Table 3:** Description of the distributions of each trait (mean, standard deviation, and range) and broad sense heritability within each environment.

	*Trait*	*DS*	*DI*	*DX*	Proportional Disease Area	One-Hot Single Threshold	Maximum Severity Average)	(Severity Average)	Weighted Average
	**Mean (s.d.)**	1.87 (2.11)	31.9 (41.4)	13.29 (21.1)	0.286 (0.128)	2.93 (2.06)	3.03 (1.16)	0.173 (0.145)	4.67 (2.95)
	**Range**	0-9	0-100	0-100	0-0.694	0-7	1-4	0-.602	0-18.69
**Heritability**	**Ames 2015**	0.59	0.43	0.50	0.64	0.50	0.52	0.42	0.62
	**Muscatine 2015**	0.79	0.54	0.83	0.39	0.30	0.28	0.23	0.32
	**Muscatine 2016**	0.55	0.44	0.53	0.54	0.59	0.35	0.64	0.58

### 3.2 RetinaNet training and evaluation

As mentioned, the RetinaNet model was utilized for the task of object detection as it reduces the data imbalance issue in the labeled dataset (**Table 1**). Our presented results are executed using a high-performance cluster with 15 nodes and a total of 60 GPUs. Each node has 64 AMD EPYC 7543 32-core CPUs and 4 NVIDIA A100-SXM GPUs. The number of epochs for the training procedure is 50 to avoid underfitting, and each six bounding boxes are fed to the model as batch size. The learning rate was assumed as 0.0001 for precise detection, and the model was initialized with the pre-trained ImageNet weights for training. The focal loss power was chosen as 2, similar to Lin et al., (2017a) study as they report it as the best value in practice. Also, the IoU threshold for finding the TP, FN and FP bounding boxes was considered 0.5 after examining several values, similar to previous works. The dropout rate was tunned and selected 0.5 for the dense layers. The threshold on the model’s confidence score to filter out detection was selected as 0.05 to ensure all the valuable bounding boxes were considered.

Results for three convolutional backbones were examined, which are ResNet-50, VGG-16 and DenseNet-121 and reported in **Table 4** by comparing mAP, accuracy, *F_1_* score, and MCC as explained in *Deep learning model*. The best metric values was noted for VGG-16 on test data (**Table 4**); therefore, this model architecture was selected for further analysis.

**Table 4:** Object detection mAP values for Train, validation and test sets for three different model backbones.

Backbone	mAP			Accuracy	*F_1_* score	MCC
	Train	Validation	Test	Test	Test	Test
**ResNet-50**	0.5936	0.3674	0.3354	0.6357	0.6449	0.4588
**DenseNet-121**	0.5890	0.3615	0.3281	0.6232	0.6191	0.4457
**VGG-16**	**0.6324**	**0.3721**	**0.3425**	**0.6548**	**0.6424**	**0.4656**

**Figure 6:**
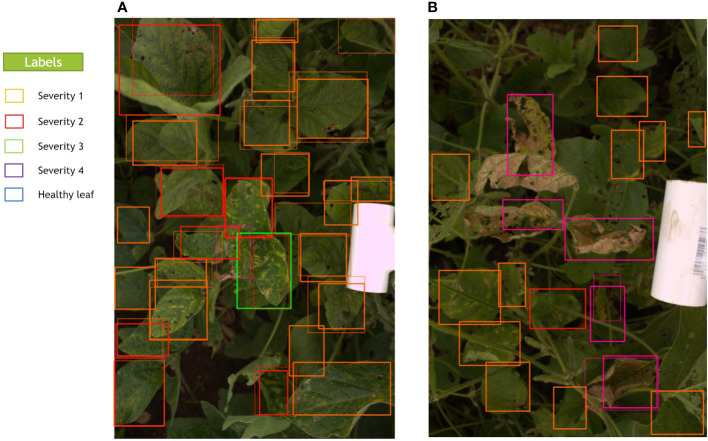
Predicted and ground truth bounding boxes for two random images **(A, B)** in the test set. Thick lines in these images show the predicted bounding boxes executed from the model, and thin lines show the ground truth bounding boxes. The model can also predict some unlabeled bounding boxes that were missed during data annotation.

**Figure 6** represents the bounding boxes the trained model predicts with convolutional backbone VGG-16 for two sample images from the test set. In this figure, the thicker bounding boxes show the predicted bounding boxes executed from the DL model, and the thinner ones indicate the ground truth bounding boxes. Moreover, to prevent having overlapped bounding boxes for one leaflet, *Non-Max Suppression* (NMS) method was applied. This implies that if two bounding boxes indicate the same leaflet and have IoU more than 0.3, the one with the lower predicted confidence score was removed. As shown in **Figure 6A**, the model predicted the majority of the labeled bounding boxes with the correct classification labels (same colors for the thick and thin bounding boxes in the image). Moreover, in some cases, some diseased leaflets were missed to be labeled; however, the model could recognize and classify them substantially (diseased leaflets on the bottom of the **Figure 6A**). This is more visible in **Figure 6B**, where so many unlabeled leaflets with Severity 4 were localized and detected by the model precisely; however, they were not annotated. Due to the same reason, if these leaflets had been labeled, superior mAP, accuracy, F-measure and MCC values would be expected from the model prediction. Generally, our results in these figures show the effectiveness of the object detection model in predicting most of the severity levels of diseased leaflets accurately. The performance of the DL model will also be justified and confirmed by analyzing the GWAS results.

**Figure 7:**
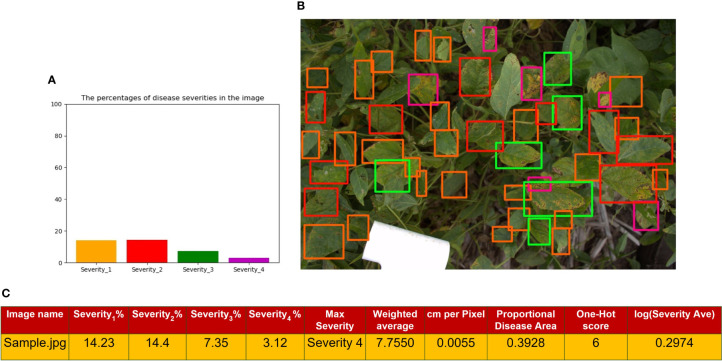
**(A)** shows a histogram representation of the total area of the bounding boxes for each class within the image **(B)** is the image output from the RetinaNet network with bounding boxes where orange is Severity 1, red is Severity 2, green is Severity3, and purple is Severity 4 **(C)** is a tabular format of the information in the histogram, where each Severity Percentage is calculated as shown in equation 9; as well as other extracted traits explained in sections 2.3.1, 2.5.2, and 2.5.3.

**Figure 7A** represents a histogram distribution of the area of the bounding boxes in **Figure 7B**, which shows a representation of the output of the RetinaNet network for a sample image. Moreover, **Figure 7C** shows the extracted traits from the image, which will be used to simulate the GWAS analysis. Severity percentages in the table were computed through equation 9. This image contains leaves with all of the severity levels. The Maximum Severity as explained in *Image-based* is the highest severity level which has a value. In this image, since we have some bounding boxes for Severity_4_, it is denoted as *Max Severity* in the table. The *cm per Pixel* was computed as mentioned in *Actual image area calculation*. Also, the *Proportional Disease Area*, *Weighted Average* and *Severity Average* were calculated with equations 10, 11, and 12, respectively. The logarithmic value for Severity Average which will be used in GWAS analysis is reported in the table. The *One-Hot score* in the table is extracted from **Table 2** as it is explained in *One-hot encoding*. All of these extracted traits will be considered as DL-traits for further analysis.

### 3.3 GWAS

Across the three programs used to perform the GWAS analysis, 46 significant SNPs, including duplicates across traits or methods, were identified with a MAF of 5% and 46 significant SNPs, including duplicates with a MAF of 1%. **Table 5** shows the distribution of SNPs that were identified across visual ratings and DL generated ratings, and the three GWAS methods when a MAF of 5% was used.

**Table 5:** Distribution of significant SNPs across programs used to run GWAS analysis (Tassel, GAPIT and SVEN) and traits with a MAF of 5% and MAF of 1%, where the MAF 1% column represents additional SNPs reported that were not reported with a MAF of 5%.

		Tassel		SVEN		GAPIT		Total	
	Trait	MAF 5%	MAF 1%	MAF 5%	MAF 1%	MAF 5%	MAF 1%	MAF 5%	MAF 1%
**Hand**									
Score	**DI**	0	0	2	1	0	0	13	17
	**DS**	1	0	5	6	0	0		
	**DX**	0	4	5	6	0	0		
**DL**									
Traits	**Weighted Average**	0	0	3	3	0	0	32	22
	**Maximum Severity**	2	0	4	3	4	2		
	**One-Hot Score with Single Threshold**	0	0	7	5	0	2		
	**Log(Severity Average)**	0	0	10	6	0	0		
	**Proportional Disease Area**	0	0	2	1	0	0		
	**Total**	3	4	38	31	4	4		

In total with a MAF of 5%, there were 13 significant SNPs found using the manually collected traits, such as disease severity, and 32 significant SNPs with image-based traits. Across methods, Tassel 5 identified 3 significant SNPs after FDR correction, SVEN reported 38 significant SNPs with a marginal inclusion probability over 0.5 and GAPIT version 3 had 5 significant SNPs after FDR correction. There were approximately 4 significant SNPs found per visually rated trait and 6 significant SNPs found per image-based trait. Significant SNPs reported from all three programs and from MAF of 5% and 1% can be found in **Table S2** and **Table S3** in the Supplementary, respectively. These SNPs are located within 21 previously reported SDS QTL on Soybase (**Figure S4**).

**Figure 8:**
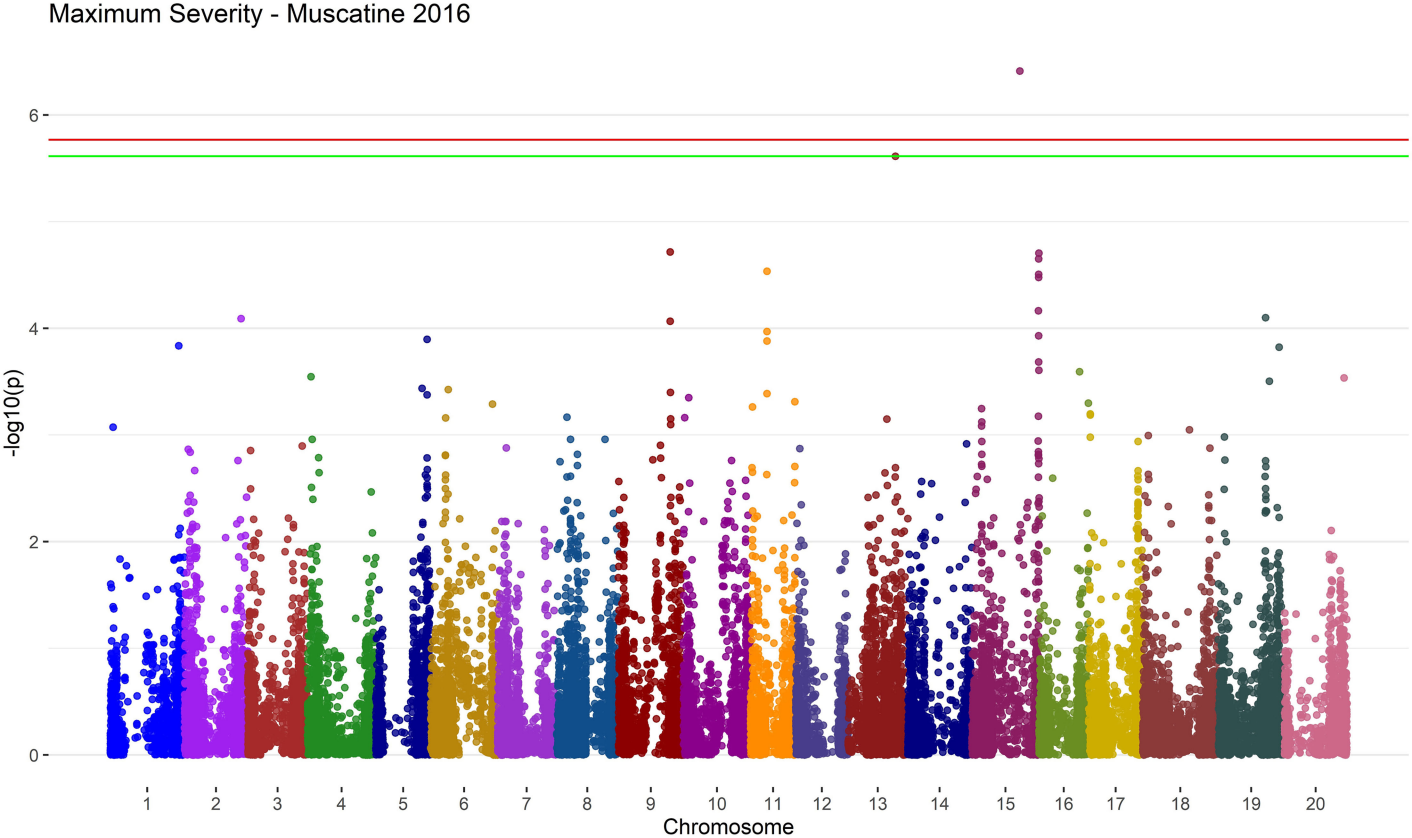
Manhattan plot of results from MLM in Tassel 5 for the Maximum Severity trait in Muscatine 2016 using a MAF of 5%. The negative log base 10 transformed p values are plotted against their position along each of the 20 chromosomes. The green line represents the FDR correction threshold and the red line represents the Bonferroni correction threshold. Significant SNPs are denoted by exceeding the FDR correction threshold. In this environment/trait combination, run with the MLM model on Tassel 5, there are two significant SNPs, one on chromosome 13 and the other on chromosome 15.

The programs showed some agreement by reporting similar SNPs as significant. In Muscatine 2016 with a MAF of 5% SVEN reported *ss715606297* as associated with Severity Average and GAPIT associated *ss715606297* with Maximum Severity. Tassel and SVEN both found an association with Maximum Severity in Muscatine 2016, with a MAF of 5%, and *ss715615734* on Chromosome 13 (**Figure 8**). The SNP *ss715615734*, is near two potential candidate genes, *Glyma.13g257100* and *Glyma.13g256500*. *Glyma.13g256500*, a COPI associated protein, is 1.1 kbp from *ss715615734*. *Glyma.13g257100* is a DnaJ-domain superfamily protein and is 56 kbp from *ss715615734*.

There was also a few SNPs near previously reported loci associated with SDS. The gene *SIK1* was previously identified as a candidate gene for SDS by Zhang et al. (2015) and is 131 kbp from *ss715584164*, a SNP reported as significant in association with DS at Muscatine in 2016 by SVEN with a MAF of 5%. With a MAF of 1%, *ss715584207* was found associated with Proportional Disease Area in Muscatine 2016 by SVEN and is 55 kbp from *SIK1*. In addition to this with a MAF of 5%, *ss715610404* was found associated with DX by SVEN and is 112 kbp from the previously reported SDS GWAS QTL on SoyBase called SDS 1-g35 (Grant et al., 2010; Wen et al., 2014).

**Table 6** lists a subset of significant SNPs and associated potential candidate genes or SDS associated QTL those SNPs are within. A potential candidate gene on Chromosome 2 is *Glyma.02g070600*, which is a NAC domain containing protein that is 19 kbp from *ss715583703*. In Muscatine 2016, *ss715583703* was reported in association with Maximum Severity with MAF of 5%. With a MAF of 1% *ss715583708* was reported as associated with One-Hot Score in Muscatine 2016 and is 25 kbp from *Glyma.02g070600*.

**Table 6:** Description of subset of significant SNPs and associated candidate genes.

**Trait- MAF%**	**Loc**	**SNP**	**Chr**	**Pos(bp)**	**GWAS**	**MIP/P**	**Canidate Gene**	**Annotation/SDS QTL**
*Maximum Severity- 5%*	Muscatine 2016	ss715583703	2	6198717	SVEN	0.607	Glyma.02g070600	NAC domain containing protein 87
*One-Hot Score - 1%*	Muscatine 2016	ss715583708	2	6242767	SVEN	0.623	Glyma.02g070600	NAC domain containing protein 87
*DS - 5%*	Muscatine 2016	ss715584164	2	9318571	SVEN	0.983	SIK1*	LRR-RLK LRR-RLK
*Proportional Disease Area - 1%*	Muscatine 2016	ss715584207	2	9509442	SVEN	0.501	SIK1*	
Maximum Severity- 5%	Muscatine 2016	ss715602785	8	8637814	GAPIT	0.0022		SDS 15-2 SDS 15-3 SDS disease incidence 20-2 SDS 16-3
*Maximum Severity- 5%*	Muscatine 2016	ss715606295	10	34735539	GAPIT	0.0025		*Potentially Novel QTL*
		ss715606297		34806626		0.0025		
		ss715606299		34885443		0.0025		
		ss715606302		34988378		0.0025		
*Severity Average- 5%*	Muscatine 2016	ss715606297	10	34806626	SVEN	0.719		
*DX - 5%*	Muscatine 2016	ss715610404	11	32865931	SVEN	0.983		SDS 1-g35*
*Maximum Severity - 5%*	Muscatine 2016	ss715615734	13	36241512	Tassel	0.032	Glyma.13g257100	DnaJ Domain
					SVEN	0.704	Glyma.13g256500	COPI associated protein

The trait, minor allele frequency used, and environment each SNP was reported associated with. Those reported by SVEN have a MIP reported (closer to one is higher likelihood of association with trait) and those reported by Tassel or GAPIT have a p value listed. The p values reported here are q-values after FDR corrections with a significance level of p < 0.05.

GWAS - program used for GWAS analysis that SNP was reported by (GAPIT, Tassel, or SVEN).

*Denotes previously reported candidate gene or QTL for SDS.

**Figure 9:**
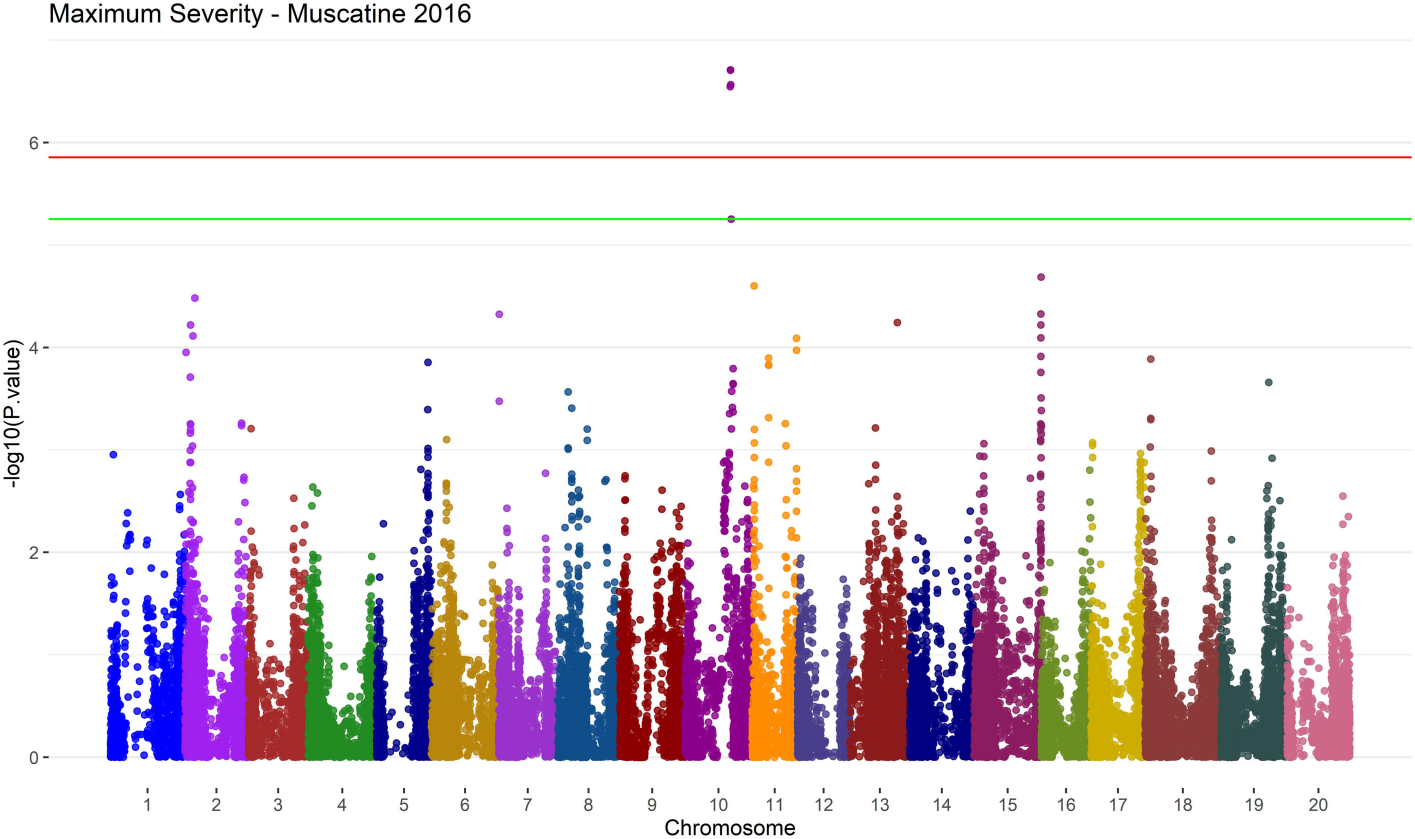
Manhattan plot of results from MLM in GAPIT version 3 for the Maximum Severity trait in Muscatine 2016 using a MAF of 5%. The negative log base 10 transformed p values are plotted against their position along each of the 20 chromosomes. The green line represents the FDR correction threshold and the red line represents the Bonferroni correction threshold. Significant SNPs can be denoted by exceeding the FDR correction threshold. In this environment/trait combination, run with the MLM model on GAPIT version 3, there is 5 significant SNPs on chromosome 10.

There were a few regions that were mostly associated with Maximum Severity and could be worth future exploration and validation. A 253 kbp region on Chromosome 10 is reported as associated with Maximum Severity by GAPIT by SNPs *ss715606295*,*ss715606297*, *ss715606299* and *ss715606302* in Muscatine 2016 with a MAF of 5% as shown in **Figure 9**.

SVEN also had a hit within this region associated with Severity Average, *ss715606297*, in Muscatine 2016 with a MAF of 5%. In Muscatine 2016 with MAF of 5%, *ss715602785* was found associated with Maximum Severity by GAPIT. This SNP is within four SDS QTL that are reported on SoyBase (Grant et al., 2010). These SDS QTL are called SDS 15-3, SDS 15-2, SDS disease incidence 20-2 and SDS 16-3 on SoyBase (Grant et al., 2010).

## 4 Discussion

In this study, a DL network, RetinaNet, was utilized to evaluate soybean canopy images taken from field trials evaluating resistance to SDS. Multiple challenges in phenotyping field image data were overcome in this study, such as having a complex background like soil, images capturing variable sizes of canopy area, and detecting diseases followed by classifying disease severity of individual leaflets. DL methods have become more popular in the last few years as a method of extracting phenotypic traits from large amounts of data (Singh et al., 2021b). Visual canopy ratings collected in the field can be subjective and difficult to classify as a canopy can be composed of areas of multiple severities that is summarized in a single rating. Our RetinaNet model isolates individual leaflets with disease symptoms in the plant canopy to classify the severity level. This allows for the extraction of traits, such as Weighted Average, that account for the variation of symptom severity within the canopy. Further validation of this method of image-based trait extraction looked at how the phenotypic data could be applied.

Here, a GWAS analysis was performed to help provide some insights into the genetic architecture of SDS resistance and compare image-based phenotypic data with manually collected visual data. Two significant SNPs were identified near a previously reported candidate gene, called *SIK1* (Zhang et al., 2017). One of these SNPs was associated with a visually collected trait, DS, and the other with an image-based trait, Proportional Disease Area. The significant SNPs were also compared to previously reported SDS QTL on Soybase. Significant SNPs associated with image-based traits are located in 11 different previously reported SDS QTL and significant SNPs associated with visually collected traits are located within 5 different previously reported SDS QTL. There were four previously reported SDS QTL that contained a significant SNP associated with an image-based traits as well as at least one significant SNP associated with a visually collected trait (**Figure S4**). This increases the confidence in the image-based traits’ ability to lead to detection of SNPs associated with SDS. SVEN, a GWAS method based on a hierarchical multi-locus model, provides further support by finding a SNP associated with DX that is near a previously reported SDS GWAS QTL, called DS 1-g35 on SoyBase (Grant et al., 2010; Wen et al., 2014).

Potentially novel candidate genes for SDS resistance were searched for to compare the amount of information obtained from using image-based traits vs manual traits. On Chromosome 13, two candidate genes were identified *Glyma.13g256500* and *Glyma.13g257100* near a SNP associated with Maximum Severity by SVEN and Tassel. *Glyma.13g256500* has been previously reported as a candidate gene for resistance to Phytophthora sojae (Li et al., 2016). While, *Glyma.13g257100*, was found to have a negative effect on the susceptibility to soybean mosaic virus when silenced (Liu and Whitham, 2013; McCabe and Graham, 2020). Both genes have been previously identified in association with disease resistance, and *Glyma.13g256500* was specifically found to have resistance to a soil-borne fungal pathogen, similar to SDS. Near *Glyma.02g070600*, a NAC domain containing protein, two SNPs were found, one associated with Maximum Severity and the other One-Hot Score. NAC transcription factors have been found to be involved in stress response and leaf senescence (Melo, 2016; Fraga et al., 2021).

An area of future investigation for a novel QTL associated with SDS resistance, based on this study, would be a region on Chromosome 10. GAPIT reported four significant SNPs within a 253 kbp region associated with Maximum Severity. SVEN also reported a significant SNP associated with Severity Average in this region. Maximum Severity also has the most significant SNPs associated with it when combining across all three programs with a MAF of 1% or 5%. Considering both MAF of 1% and 5% the next comparable traits are DS, Severity Average and DX.

Manual phenotyping can be a labor and time intensive task. GWAS studies commonly consists of large panels of accessions with data collected in multiple environments. RetinaNet, a one-stage object detector, leads to faster processing time of images. The time required to phenotype individual plots *via* imaging and processing is greatly reduced and allows for the collection of larger data sets. Using this method of image-based phenotyping could aid in the collection of data from larger GWAS panels or within a larger scale breeding program for disease testing. Within the identification, classification, quantification, and prediction (ICQP) process of using DL for plant phenotyping (Singh et al., 2018) our method involves three steps, ICQ. The RetinaNet model constructed in this paper focuses on improving quantification. In each image the severity of leaflets are quantified and that quantification is then used in analysis. A next step could be combining DL methods like the one used in this study and by (Nagasubramanian et al., 2020) to identify diseased leaves or leaflets, classify them to a disease, and then quantify the severity of the canopy. Developing a model such as this could then be used in applications for research, breeding, and education. Image data could be collected *via* rovers, unmanned aerial vehicles, or phones for use by researchers, farmers, or breeding programs.

In this study, a method for leveraging image data to extract potentially meaningful traits is presented and compared to manually collected visual traits. Image based traits were validated by detecting regions near previously reported SDS loci. They were then evaluated to aid in the search of candidate genes for resistance to SDS. There were several genes found that could potentially offer resistance from the image-based traits. Some of the image-based traits appear to be more informative than others in terms of association to SDS resistance. The framework proposed in this study could help develop similar models for other diseases in a variety of crops that could then be deployed across multiple platforms (e.g., drones) to aid in the high throughput characterization of disease severity levels.

## Data availability statement

The raw data supporting the conclusions of this article will be made available by the authors, without undue reservation.

## Author contributions

AR and FF contributed equally to proposing ideas, analyzing, implementing and programming the methods. AS conceptualized the original experiment, and AS and SS provided the framework for the research and analysis, as well as led the project management. JZ collected the disease ratings and images. AS, SS, BG, AKS, and SD provided technical inputs for the approach, analysis, and interpretation. DSM and AKS provided resources for disease nurseries. All authors evaluated the results, wrote, read, and reviewed the manuscript. All authors contributed to the article and approved the submitted version.

## Funding

This work was supported by AI Institute for Resilient Agriculture (USDA-NIFA #2021-67021-35329), COALESCE: COntext Aware LEarning for Sustainable CybEr-Agricultural Systems (NSF CPS Frontier # 1954556), FACT: A Scalable Cyber Ecosystem for Acquisition, Curation, and Analysis of Multispectral UAV Image Data (USDA-NIFA #2019-67021-29938), Smart Integrated Farm Network for Rural Agricultural Communities (SIRAC) (NSF S&CC #1952045), and USDA CRIS Project IOW04714. Support was also provided by R F Baker Center for Plant Breeding, Iowa Soybean Association, and Plant Sciences Institute.

## Acknowledgments

We thank team members of AKS and AS groups for help in imaging and data collection. We thank Jae Brungardt and Brian Scott for their assistance in establishing the disease nursery and field experimentation, respectively.

## Conflict of interest

The authors declare that the research was conducted in the absence of any commercial or financial relationships that could be construed as a potential conflict of interest.

## Publisher’s note

All claims expressed in this article are solely those of the authors and do not necessarily represent those of their affiliated organizations, or those of the publisher, the editors and the reviewers. Any product that may be evaluated in this article, or claim that may be made by its manufacturer, is not guaranteed or endorsed by the publisher.
