# Supplementary material for: Deep learning-based phenotyping for genome wide association studies of sudden death syndrome in soybean
Source: Front Plant Sci. 2022 Oct 21;13:966244. doi: 10.3389/fpls.2022.966244 (PMC9634489; doi:10.3389/fpls.2022.966244)
Supplement: Supplementary file 1 [file DataSheet_1.pdf]

Supplementary Material

1 RETINANET DETAILS

1.1 Backbone Network

Detecting the small objects needs an accurate network such as Feature Pyramid Network. In FPN, the model is composed of bottom-up and up-down paths. The convolutional neural network extracts the features of an image in the bottom-up pathway. In this work, VGG16 is used as a CNN model to develop the feature maps. In the FPN model, the image resolution decreases in an up-down way; however, each layer's semantic value increases. In the up-down path, the higher resolution layers from a semantic layer are constructed. The location of the objects can extract by adding lateral connections between feature maps and up-down layers. In our case, 256 channels are considered for all pyramid levels. Moreover, as reported in the Retinanet article, the pyramids with levels 3 to 7 are used.Detecting the small objects needs an accurate network such as Feature Pyramid Network. In FPN, the model is composed of bottom-up and up-down paths. The convolutional neural network extracts the features of an image in the bottom-up pathway. In the FPN model, the image resolution decreases in an up-down way; however, each layer's semantic value increases. In the up-down path, the higher resolution layers from a semantic layer are constructed. The location of the objects can extract by adding lateral connections between feature maps and up-down layers.

1.2 Subnetworks

Two subnetworks are applied in the Retinanet model, which are Classification Subnet and Box Regression Subnet. In the classification subnet, for each of the anchor boxes and object classes, the probability of object existence at each spatial position is predicted. A fully convolutional network is attached to each FPN level for the classification task. This convolutional layer consists of four 3*3 Conv layers with 4C (C is the number of channels) filters. These layers are followed by a 3*3 convolutional layer and sigmoid activation to give each anchor the class probability. In this work, for each spatial position, nine anchor boxes are considered.

The box regression network is a parallel network with the classification network, which like the classification task, another fully convolutional layer is designed to regress the offset between each anchor box and a nearby ground truth object. For the network's output, in each spatial position and each anchor box, the network has four outputs that predict the relative offset. More information about these subnets is mentioned in (Lin et al., 2017). Although these two subnets possess the same structures, they use separate parameters.

1.3 Focal Loss

As mentioned in section above, focal loss is designed to address the imbalance problems between foreground and background classes. Focal loss is the improved version of cross-entropy loss. The definition of the class entropy loss is as follows:

$$CE(p,y) = \begin{cases} -log(p) & \text{if } y = 1 \\ -log(1-p) & Otherwise \end{cases} \tag{S1}$$

For the sake of simplicity, variable p_t is defined as:

$$p_t = \begin{cases} p & \text{if } y = 1 \\ 1 - p & Otherwise \end{cases} \tag{S2}$$

With this definition of p_t, the cross entropy loss function would be as follow:

$$CE(p_t) = -log(p_t) \tag{S3}$$

Therefore, if a modulating factor multiplies to cross-entropy loss and a tunable element $(\lambda > 0)$ is defined, the focal loss would be as follows in:

$$FL(p_t) = -(1 - p_t)^{\lambda} log(p_t) \tag{S4}$$

This loss has two properties: First, if a class is misclassified, the term$(1 - p_t)$ closes to 1, so, in comparison to the classes that classified correctly, this loss is so higher. Therefore, the objects in the foreground can be predicted with more emphasis on them. Second, if the parameter λ increases, the objects with easy correct predictions would be decreased for training.

REFERENCES

Lin, T.-Y., Goyal, P., Girshick, R., He, K., and Dollár, P. (2017). Focal loss for dense object detection. In *Proceedings of the IEEE international conference on computer vision*. 2980–2988

Table S1. Distribution of number of images originally collected, number of images with less than 85% canopy in the image foreground, and number of images remaining in the dataset after manual evaluation with each environment and across the whole dataset.

	Original Number of Photos	Number of photos selected for removal with 85% threshold	Number of remaining images
Muscatine 2015	1034	189	877
Ames 2015	1081	167	967
Muscatine 2016	1046	42	928
Total	3161	398	2772

Table S2. List of all significant SNPs reported based on MIP of greater than 0.5 or a FDR correction with a significance threshold of p = 0.05 from all three programs with a MAF of 5%. The p values reported here are q-values after FDR corrections.
GWAS Method - the program used for analysis that the SNP was reported by

SNP	Chromosome	Position	Trait	Environment	MIP or p	GWAS Method
ss715583146	2	4845732	One Hot Score	Muscatine 2016	0.620654001	SVEN
ss715583703	2	6198717	Maximum Severity	Muscatine 2016	0.607137864	SVEN
ss715584022	2	8589539	Proportional Disease Area	Muscatine 2015	0.510679954	SVEN
ss715584164	2	9318571	DS	Muscatine 2016	0.983261773	SVEN
ss715584376	3	1142234	Severity Average	Ames 2015	0.648902733	SVEN
ss715584758	3	2160604	One Hot Score	Muscatine 2016	0.635251287	SVEN
ss715591100	5	35069626	Severity Average	Muscatine 2016	0.718785483	SVEN
ss715591411	5	37297357	DS	Ames 2015	0.67724841	SVEN
ss715591556	5	38099881	One Hot Score	Ames 2015	0.83786337	SVEN
ss715591558	5	38124189	One Hot Score	Muscatine 2016	0.794090766	SVEN
ss715594085	6	30002101	DS	Ames 2015	0.583371607	SVEN
ss715594240	6	36084443	DI	Ames 2015	0.541440991	SVEN
ss715594899	6	48468113	DX	Muscatine 2015	0.949129638	SVEN
ss715598472	7	6349120	DS	Muscatine 2015	0.967430764	SVEN
ss715599303	8	11602284	Severity Average	Muscatine 2016	0.728483386	SVEN
ss715602305	8	45132778	Severity Average	Ames 2015	0.999788203	SVEN
ss715605323	9	6653597	Weighted Average	Ames 2015	0.52172127	SVEN
ss715603767	9	38963114	Proportional Disease Area	Muscatine 2016	0.564311434	SVEN
ss715606972	10	4021337	Severity Average	Muscatine 2016	0.70629561	SVEN
ss715608495	10	7132568	DS	Muscatine 2016	0.000527195	Tassel
ss715606295	10	34735539	Maximum Severity	Muscatine 2016	0.002513086	GAPIT
ss715606297	10	34806626	Maximum Severity	Muscatine 2016	0.002513086	GAPIT
ss715606297	10	34806626	Severity Average	Muscatine 2016	0.718973437	SVEN
ss715606299	10	34885443	Maximum Severity	Muscatine 2016	0.002513086	GAPIT
ss715606302	10	34988378	Maximum Severity	Muscatine 2016	0.002513086	GAPIT
ss715606313	10	35440548	Maximum Severity	Muscatine 2016	0.039430656	GAPIT
ss715609163	11	1530847	Maximum Severity	Muscatine 2016	0.577003408	SVEN
ss715609899	11	15221133	Severity Average	Muscatine 2016	0.738211794	SVEN
ss715609954	11	27837870	Maximum Severity	Ames 2015	0.661351282	SVEN
ss715610613	11	33656255	One Hot Score	Ames 2015	0.952940134	SVEN
ss715612158	12	32264206	Weighted Average	Muscatine 2016	0.602206862	SVEN
ss715614618	13	28397668	DS	Ames 2015	0.54170897	SVEN
ss715614857	13	29790648	DX	Muscatine 2015	0.854210784	SVEN
ss715614959	13	30322045	DI	Muscatine 2015	0.652078324	SVEN
ss715615734	13	36241512	Maximum Severity	Muscatine 2016	0.703912488	SVEN
ss715615734	13	36241512	Maximum Severity	Muscatine 2016	0.03442387	Tassel
ss715619831	14	7089189	DX	Muscatine 2015	0.90762608	SVEN
ss715619199	14	46168096	Severity Average	Muscatine 2016	0.719725142	SVEN
ss715619200	14	46171616	Weighted Average	Muscatine 2016	0.666228703	SVEN
ss715623031	15	7567313	Severity Average	Muscatine 2016	0.720187125	SVEN
ss715621659	15	36882475	Maximum Severity	Muscatine 2016	0.010979962	Tassel
ss715623929	16	28527320	DX	Muscatine 2016	0.71050877	SVEN
ss715623977	16	28785225	One Hot Score	Ames 2015	0.638214902	SVEN
ss715628225	17	7913612	DX	Muscatine 2016	0.671136433	SVEN
ss715627315	17	37876463	Severity Average	Muscatine 2016	0.576640125	SVEN
ss715631024	18	45051913	One Hot Score	Ames 2015	0.657134542	SVEN

Table S3. List of all significant SNPs reported based on MIP of greater than 0.5 or a FDR correction with a significance threshold of p = 0.05 from all three programs with a MAF of 1%. The p values reported here are q-values after FDR corrections.
GWAS Method - the program used for analysis that the SNP was reported by

SNP	Chromosome	Position	Trait	Environment	MIP or p	GWAS Method
ss715578520	1	1475349	Severity Average	Muscatine 2016	0.929688185	SVEN
ss715579124	1	36992932	DX	Ames 2015	0.502531092	SVEN
ss715583595	2	5430492	Severity Average	Muscatine 2016	0.923743626	SVEN
ss715583708	2	6242767	One Hot Score	Muscatine 2016	0.623186909	SVEN
ss715584207	2	9509442	Proportional Disease Area	Muscatine 2015	0.500507635	SVEN
ss715580930	2	10618540	DS	Muscatine 2016	0.928330094	SVEN
ss715585498	3	3691222	Severity Average	Ames 2015	0.734467585	SVEN
ss715586730	3	4939933	One Hot Score	Muscatine 2016	0.840026631	SVEN
ss715586755	3	5044674	DS	Muscatine 2015	0.531035642	SVEN
ss715590782	5	32783263	One Hot Score	Ames 2015	0.029681238	GAPIT
ss715591223	5	35911860	DS	Muscatine 2016	0.583524175	SVEN
ss715592174	5	38945499	One Hot Score	Ames 2015	0.701671915	SVEN
ss715592051	5	39418662	Weighted Average	Muscatine 2016	0.592770207	SVEN
ss715591649	5	41780982	DS	Ames 2015	0.697402776	SVEN
ss715593940	6	2352768	One Hot Score	Muscatine 2016	0.76656804	SVEN
ss715598604	7	7359007	DX	Muscatine 2015	0.829669659	SVEN
ss715596313	7	14982826	DS	Muscatine 2015	0.992313155	SVEN
ss715602785	8	8637814	Maximum Severity	Muscatine2016	0.002202954	GAPIT
ss715600444	8	20047987	Maximum Severity	Muscatine 2016	0.985912667	SVEN
ss715602353	8	46199621	DX	Muscatine 2016	0.041931516	Tassel
ss715603378	9	27657975	Severity Average	Ames 2015	0.995975561	SVEN
ss715604609	9	45672663	Weighted Average	Ames 2015	0.524103794	SVEN
ss715608495	10	7132568	DS	Muscatine 2016	0.000611684	Tassel
ss715606295	10	34735539	Maximum Severity	Muscatine2016	0.002202954	GAPIT
ss715606297	10	34806626	Maximum Severity	Muscatine2016	0.002202954	GAPIT
ss715606299	10	34885443	Maximum Severity	Muscatine2016	0.002202954	GAPIT
ss715606302	10	34988378	Maximum Severity	Muscatine2016	0.002202954	GAPIT
ss715606313	10	35440548	Maximum Severity	Muscatine2016	0.036818365	GAPIT
ss715609003	11	1354190	DX	Muscatine 2016	0.041931516	Tassel
ss715610404	11	32865931	DX	Muscatine 2016	0.983119372	SVEN
ss715614023	13	19858948	Maximum Severity	Muscatine2016	0.049502853	GAPIT
ss715614672	13	28622330	DX	Muscatine 2016	0.041931516	Tassel
ss715616510	13	44046219	One Hot Score	Ames 2015	0.032184586	GAPIT
ss715618797	14	4389990	DS	Ames 2015	0.550479505	SVEN
ss715619825	14	7063747	DX	Muscatine 2015	0.658322052	SVEN
ss715619923	14	7955420	DI	Muscatine 2015	0.656455927	SVEN
ss715618238	14	34129436	Maximum Severity	Muscatine 2016	0.995929011	SVEN
ss715618238	14	34129436	Severity Average	Muscatine 2016	0.996132307	SVEN
ss715620209	15	9445842	DX	Muscatine 2015	0.715969389	SVEN
ss715621600	15	34986377	Weighted Average	Muscatine 2016	0.635817906	SVEN
ss715621659	15	36882475	Maximum Severity	Muscatine 2016	0.011714453	Tassel
ss715627874	17	3924278	Maximum Severity	Muscatine 2016	0.541873244	SVEN
ss715632923	18	9495115	DX	Muscatine 2016	0.888120266	SVEN
ss715629935	18	22035302	Severity Average	Muscatine 2016	0.760357914	SVEN
ss715632427	18	57152735	DX	Muscatine 2016	0.041931516	Tassel
ss715634535	19	37009318	One Hot Score	Ames 2015	0.774219666	SVEN

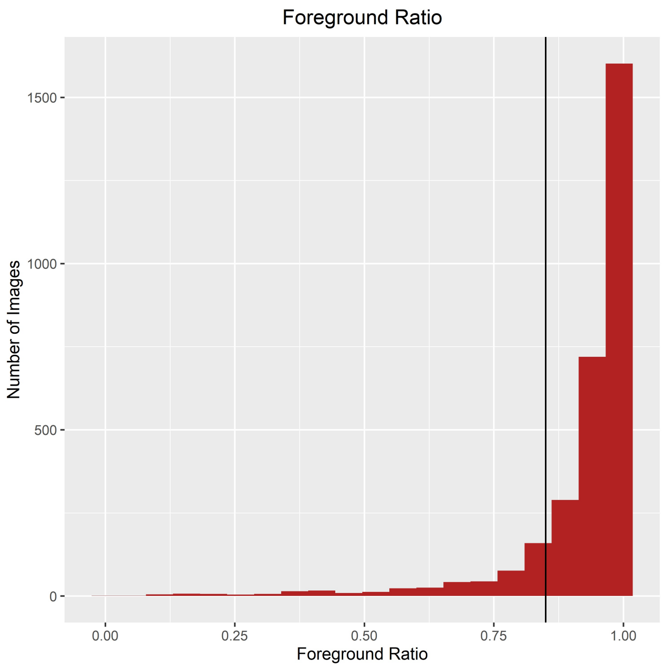

Figure S1. Histogram of distribution of foreground ratio distribution within the dataset. The black line shows the cutoff threshold of 85%.

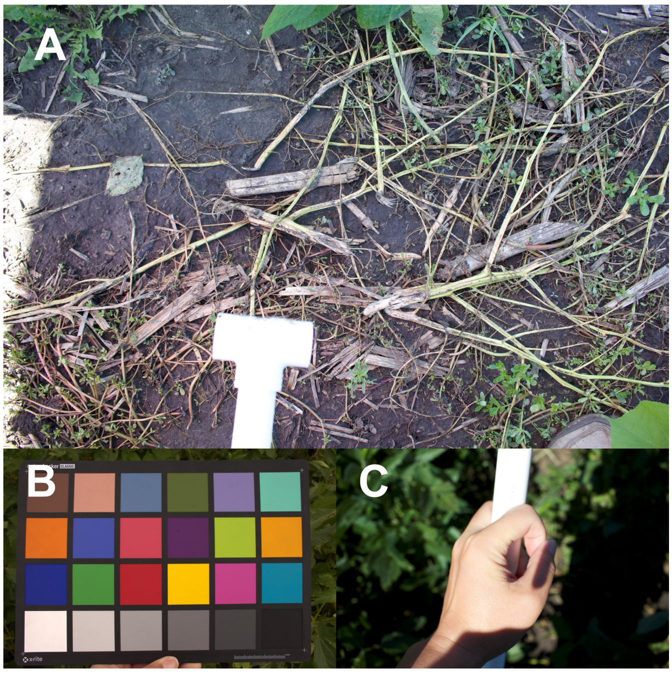

Figure S2. Example of images removed from original image dataset. (A) is an example of a plot with no canopy, (B) is an example of an image of a color card, and (C) is an image that was used to track location within the field while imaging.

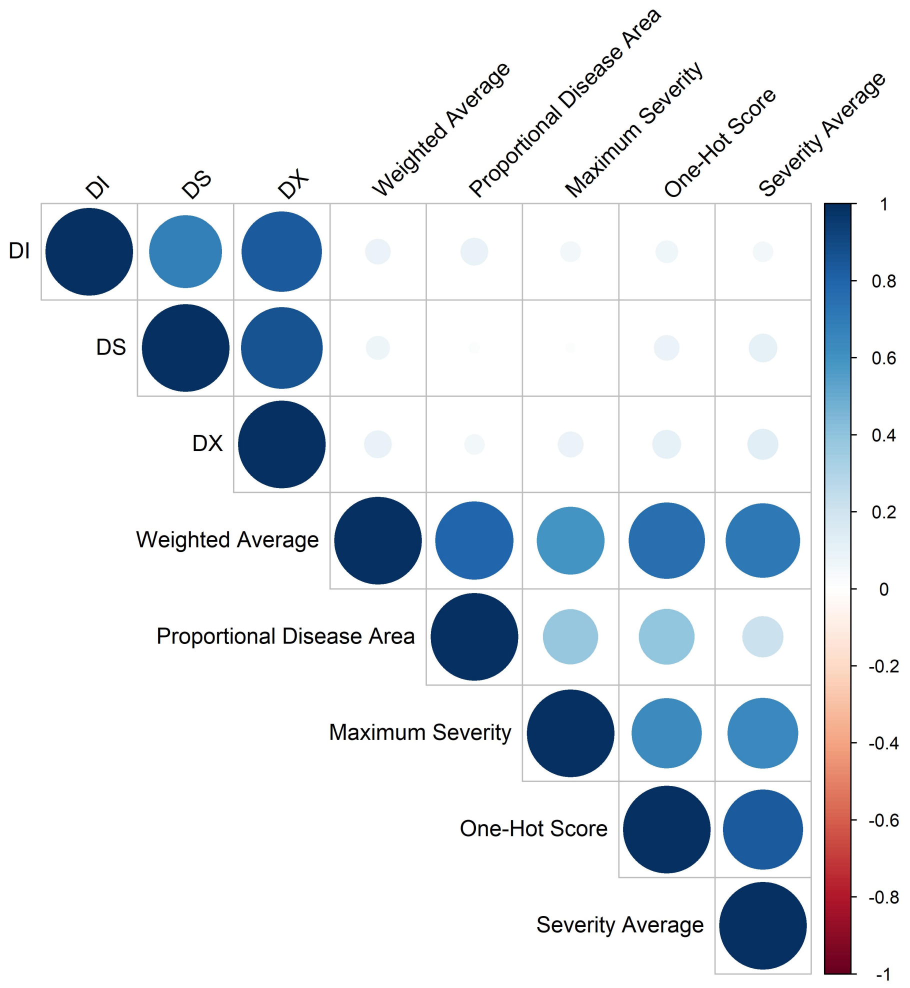

Figure S3. Correlation matrix of the visually collected traits and DL associated traits extracted from images. Disease index (DX) is calculated from disease incidence (DI) and disease severity (DS). DL associated traits are independent of each other. Stronger correlations seen within visually collected traits and DL associated traits separately with lower correlation between the groups.

LG	*Trait*	*Group*	*SNP*	*Env*	*MAF %*	*Chr*	*Pos*	*SDS QTL*
D1b	One Hot Score		ss715583146	Muscatine 2016	5	2	4845732	SDS 13-4
	Proportional Disease Area		ss715584022	Muscatine 2015	5	2	8589539	SDS 13-5
	DS		ss715584164	Muscatine 2016	5	2	9318571	SDS 13-5
A1	Severity Average		ss715591100	Muscatine 2016	5	5	35069626	SDS 15-8
	DS		ss715591223	Muscatine 2016	1	5	35911860	SDS 15-8
	DS		ss715591411	Ames 2015	5	5	37297357	SDS 15-8,SDS 14-4
	One Hot Score		ss715591556	Ames 2015	5	5	38099881	SDS 14-4
	One Hot Score		ss715591558	Muscatine 2016	5	5	38124189	SDS 14-4
C2	One Hot Score		ss715593940	Muscatine 2016	1	6	2352768	SDS 13-14
	DS		ss715594085	Ames 2015	5	6	30002101	SDS 11-1,SDS 16-6
	DI		ss715594240	Ames 2015	5	6	36084443	SDS 11-1,SDS 16-6
A2	Maximum Severity		ss715602785	Muscatine2016	1	8	8637814	SDS 15-3,SDS 16-3
	Severity Average		ss715599303	Muscatine 2016	5	8	11602284	SDS 16-3
K	Severity Average		ss715603378	Ames 2015	1	9	27657975	SDS 16-1,SDS 18-3
O	Severity Average		ss715606972	Muscatine 2016	5	10	4021337	SDS 14-8
	DS		ss715608495	Muscatine 2016	1,5	10	7132568	SDS 16-7
B1	DX		ss715609003	Muscatine 2016	1	11	1354190	SDS 13-8
	Maximum Severity		ss715609163	Muscatine 2016	5	11	1530847	SDS 13-8
F	Maximum Severity		ss715614023	Muscatine 2016	1	13	19858948	SDS 17-2
B2	DS		ss715618797	Ames 2015	1	14	4389990	SDS 14-10
	DX		ss715619825	Muscatine 2015	1	14	7063747	SDS 14-10
	DX		ss715619831	Muscatine 2015	5	14	7089189	SDS 14-10,SDS disease index 21-1
	DI		ss715619923	Muscatine 2015	1	14	7955420	SDS 14-10,SDS disease index 21-1
E	Weighted Average		ss715621600	Muscatine 2016	1	15	34986377	SDS disease index SDS 21-2
	Maximum Severity		ss715621659	Muscatine 2016	1,5	15	36882475	SDS disease index SDS 21-2
D2	Maximum Severity		ss715627874	Muscatine 2016	1	17	3924278	SDS 17-3
G	Severity Average		ss715629935	Muscatine 2016	1	18	22035302	SDS 11-5

Figure S4. Subset of significant SNPs that are located with a previously reported SDS QTL on Soybase.
LG - linkage group SNP and SDS QTL are located in
Group - visual representation of cluster of SNPs found within the same SDS QTL
MAF% - minor allele frequency used in filtering before GWAS analysis that SNP was reported with
SDS QTL - name of QTL based on Soybase nomenclature
